# Ductility-strength and strength-ductility relations for a constant yield displacement seismic design procedure

**DOI:** 10.1007/s10518-023-01683-1

**Published:** 2023-04-14

**Authors:** Andréia H. A. da Silva, Anastasios Tsiavos, Božidar Stojadinović

**Affiliations:** Department of Civil, Environmental, and Geomatic Engineering, ETH Zürich, Stefano-Francini-Platz 5, 8093 Zürich, Switzerland

**Keywords:** Constant yield displacement design, Displacement-based design, Seismic design

## Abstract

The modern engineering approach to design of structures exposed to rare but intense earthquakes allows for their inelastic response. Models and tools to rapidly but accurately assess the extent of the inelastic response of the structure and control its performance are, therefore, essential. We develop a closed-form $\upmu -R^{*}-S_{d,y}$ relation between the ductility $\upmu$ and the strength reduction factor R*, as well as its approximate inverse R*-$\upmu$-*S*_d,y_ relation, both functions of the SDOF oscillator yield displacement *S*_d,y_, not its vibration period *T*. The fundamental vibration period of the structure varies during the iterative design process focused on modifying its strength. However, the yield displacement of the structure is practically invariant with respect to the strength of the structure, as it depends primarily on its geometry and material properties. We use these relations to formulate a constant yield displacement seismic design procedure and exemplify it. Noting the structure of the developed relations, we use dimensional analysis to formulate a version of the ductility-strength and strength-ductility relations that are dimensionless and independent of the seismic hazard intensity. These novel, dimensionless master relations are the $\upmu$-R*-*H/B*-$\upkappa$ ductility-strength and the R*-$\upmu$-*H/B*-$\upkappa$ strength-ductility relations.

## Introduction

The seismic design of a structure following modern codes (CEN 2005; SIA 2003; ASCE 2017) is an iterative process. It usually starts with only a sketch of the structural system, typically its rough geometric layout, distribution of its masses and mechanical properties of the materials, as well as the design-basis seismic hazard, usually specified by an elastic seismic design spectrum. Notably, if the geometry and the material properties of the structure are maintained through the design iteration, the yield displacement of the structure remains practically invariant (Priestley 2000; Aschheim 2002; Tsiavos and Stojadinović 2019). Following this premise, Aschheim and Black (2000) introduced the yield point spectrum (YPS) method for seismic design and evaluation of structures (Aschheim et al. 2019). The YPS design process starts with a realistic estimate of the yield displacement of the structure and its displacement ductility capacity, followed by iterations on the yield strength of the structure until convergence while keeping its yield displacement invariable. This design approach leads to a better first guess of the structural parameters and fewer design iterations compared to other force- or displacement-based seismic design methods that start by estimating the vibration period of the structure and keep it invariant during the design iterations (Hernández-Montes and Aschheim 2019).

Modern seismic design methods utilize estimates of maximum inelastic displacements of the structure. These are usually obtained from strength-ductility relations or estimates of the ratio of maximum inelastic and elastic displacements of a SDOF oscillator. To unite the concepts of yield displacement invariability with respect to the SDOF oscillator strength and the strength-ductility relations, Tsiavos and Stojadinović (2019) proposed the $\mu$-$R^*$-*H*/*B* relation. Tsiavos and Stojadinović parameterized the relation between the displacement ductility and the yield displacement (therein a function of height *H* and aspect ratio *H*/*B*) of the SDOF oscillator using the strength reduction factor $R^*$, associated with the Constant Yield Displacement (CYD) assumption, labeled so to emphasize the difference between it and the conventional strength reduction factor *R* associated with the Constant Period (CP) assumption, as shown in Fig. 3 of Tsiavos and Stojadinović (2019).

Herein, we further develop the $\mu$-$R^*$-*H*/*B* relation under the assumption that the yield displacement of a SDOF oscillator remains constant when its strength changes, the CYD framework. First, we represent the elastic seismic response of a SDOF oscillator associated with the design-basis seismic hazard using the design spectrum format common in modern seismic design codes. Second, we use the $C_R$-*R*-*T* relation between the SDOF oscillator period *T*, the strength reduction factor *R* and the inelastic displacement amplification ratio $C_R$ developed by Ruiz-García and Miranda (2007) to estimate the maximum displacement of an inelastic SDOF oscillator with respect to the elastic displacement of the SDOF oscillator of the same period. Crucially, we are able to use the $C_R$-*R*-*T* relation to further develop the $\mu$-$R^*$-*H*/*B* relation by relating $R^*$ to *R* and $S_{d,y}$ to *T*, thus adopting the final results of already conducted extensive inelastic seismic response analyses. Third, we derive a closed-form, hazard-dependent ductility-strength relation $\mu$-$R^*$–$S_{d,y}$ and its approximate inverse, the strength-ductility $R^*$–$\mu$-$S_{d,y}$ relation. Then, we demonstrate how to use this strength-ductility relation for seismic design of structures while remaining fully in the CYD framework, without referencing the period of the structure.

Furthermore, to generalize, we employ dimensional analysis to investigate how the CYD ductility-strength and strength-ductility relations depend on the design-basis seismic hazard. We derive $\mu$-$R^*$-*H*/*B*-$\kappa$ and $R^*$-$\mu$-*H*/*B*-$\kappa$ relations using the characteristic length ratio $\kappa$, defined as the ratio of the size of the SDOF oscillator to a length quantity that characterizes the design-basis seismic hazard. We demonstrate that these relations are self-similar with respect to measures of seismic hazard intensity, and thus hazard-intensity-independent. The $R^*$-$\mu$-*H*/*B*-$\kappa$ strength-ductility and the $\mu$-$R^*$-*H*/*B*-$\kappa$ ductility-strength relations are master equations that that lay the foundation for the displacement-based seismic design in the CYD framework. The main focus of this study is on the demonstration of the use of these relations for the seismic design of structures. An example showing a potential extension of the use of these relations for the seismic evaluation of structures is included in the Appendix of this paper. However, the demonstration of the detailed procedure for the use of these relations for the seismic evaluation of structures is beyond the scope of this paper. We conclude by emphasizing the advantages of these self-similar, hazard-intensity independent strength-ductility and ductility-strength relations, which provide higher stability in the design procedure and a more realistic first guess of the yield point of the structure, leading to fewer design iterations (Hernández-Montes and Aschheim 2019).

## Preliminaries

In this section, we present the main concepts used to derive the $\mu$-$R^*$-$S_{d,y}$ and $R^*$-$\mu$-$S_{d,y}$ relationships.

### Seismic design spectra

**Fig. 1:**
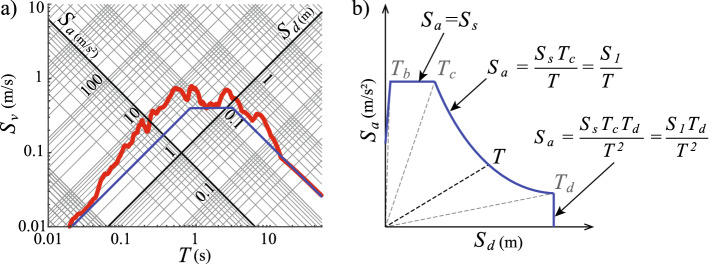
**a** Ground motion spectrum in the logarithmic tripartite format of the North–South component of the 1940 El Centro ground motion record; **b** piecewise definition of the seismic design spectrum in the ADRS format

The equation of motion of an elastic SDOF oscillator subjected to a ground motion excitation $\ddot{u}_g$ is:

1 $$\begin{aligned} \ddot{u} + 2 \zeta \omega {\dot{u}} + \omega ^2 u = -\ddot{u}_g \end{aligned}$$

where *u* is the displacement of the oscillator, $\zeta$ is its viscous damping ratio, and $\omega$ is its natural frequency. The elastic ground motion response spectrum is the plot of the maximum elastic displacements of SDOF oscillators with different natural vibration periods to a given ground motion excitation. The elastic response spectrum of a (non-pulse-like) ground motion plotted in a logarithmic tripartite graph can be delineated by a trapezoid (Newmark and Hall 1982), e.g. the 1940 El Centro ground motion record shown in Fig. 1a. The sides of the trapezoid partition the spectrum into regions characterized by pseudo-spectral acceleration $S_a$, pseudo-spectral velocity $S_v$ and spectral displacement $S_d$ values (Chopra 2016). Note that there is a relationship between the pseudo-spectral quantities and the spectral displacement (Chopra 2016):

2 $$\begin{aligned} S_d(T)= \left( \frac{T}{2\pi } \right) S_{v}(T) =\left( \frac{T}{2\pi } \right) ^2 S_{a}(T) \end{aligned}$$

and herein we omit the term pseudo when referring to $S_a$ and $S_v$.

The trapezoid-delineated response spectrum shape is the basis for the common definition of the code seismic design spectra. Usually, the code design spectra have four regions: (i) the very-short-period region, in which the spectral acceleration increases as the period increases, (ii) the short-period region, in which the spectral acceleration is constant, (iii) the medium-period region, in which the spectral velocity is constant, and (iv) the long-period region, in which the spectral displacement is constant. This approach is convenient as the design spectrum is defined as a piecewise function of the period with only a few parameters (Fig. 1b). The thresholds between the four regions of the design spectrum are given by characteristic period values (Fig. 1b). These are: $T_b$, defining the transition between very-short and short-period regions, $T_c$, defining the transition between short-period and medium-period regions, and $T_d$, defining the transition between medium-period and long-period regions.

The modern codes use two spectral values to define the elastic seismic design spectrum: the spectral acceleration in the short-period region $S_s$ and the spectral velocity in the medium-period region $S_1$. Therefore, we simplify the elastic design spectrum by neglecting the very-short-period spectral region and extending the short-period, constant-spectral-acceleration region for all SDOF oscillators with periods shorter than the corner period $T_c$. Note that the corner period $T_c = S_1/S_s$ and the corner period $T_d$ may be specified explicitly or implicitly, for example by requiring that structures have a minimum seismic base shear strength. Also, the natural vibration period of the SDOF oscillator $T=2\pi \sqrt{S_{d}/S_{a}}$ at any point of the elastic design spectrum, and it is constant at a radial, constant-period line (Fig. 1b).

**Fig. 2:**
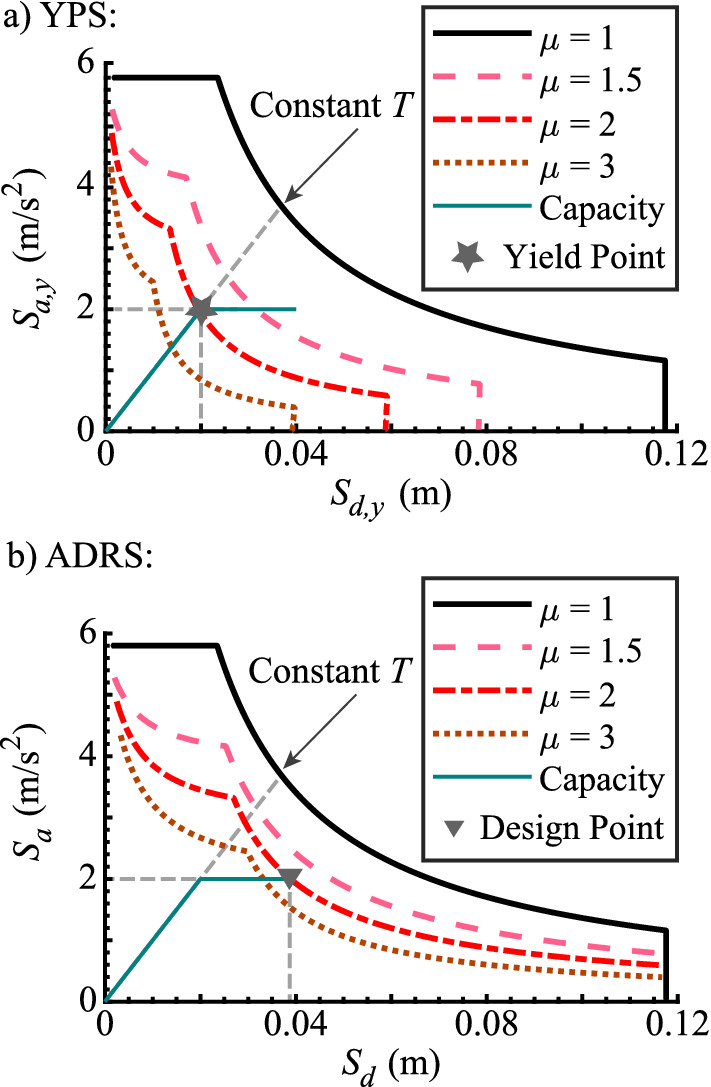
Seismic design spectra in the YPS (**a**) and the ADRS (**b**) formats and the same capacity curve of a SDOF oscillator surrogate of the structure with its Yield Point (**a**) and Design point (**b**)

The notion of an elastic response spectrum can be extended to SDOF oscillators that respond inelastically. The equation of motion of an inelastic SDOF oscillator is (Chopra 2016):

3 $$\begin{aligned} \ddot{\mu } + 2 \zeta \omega {\dot{\mu }} + \omega ^2 {\tilde{f}}_s(\mu ) = - \omega ^2 \ddot{u}_g / S_{a,y} \end{aligned}$$

where $\mu$ is the displacement ductility, and ${\tilde{f}}_s(\mu )$ is the force-deformation relationship normalized by the yield strength and yield displacement of the SDOF oscillator. Notably, the ground motion excitation $\ddot{u}_g$ is scaled by the yield spectral acceleration $S_{a,y}=F_y/m$, the SDOF oscillator yield strength normalized by its mass. The inelastic response spectrum is a plot of maximum inelastic displacements of SDOF oscillators with a yield spectral acceleration $S_{a,y}$ and varying natural vibration periods *T* to a given ground motion excitation. The inelastic design spectra for four different displacement ductility values in YPS and ADRS formats are shown in Fig. 2.

### C_R_-R-T relation

Ruiz-García and Miranda (2007) investigated the relation between the maximum displacement of an elastic SDOF oscillator with a given natural vibration period *T* and the maximum displacement of inelastic SDOF oscillators with the same vibration period, but different yield strengths, to the same ground motion excitation. To do this, they defined $C_R$ as the ratio of the maximum inelastic to the corresponding elastic SDOF oscillator displacements under the given ground motion excitation. Starting from Eq. 3, one can show that $C_R = \mu /R$ (Chopra 2016).

Ruiz-Garcia and Miranda used a suite of 240 earthquake records to generate the relevant SDOF oscillator response data. They described the central tendency

4 $$\begin{aligned} {\tilde{C}}_R = \left\{ \begin{matrix} 1 &{} &{} R \le 1\\ 1 + \frac{R-1}{\theta _1 T^{\theta _2}} &{} &{} R > 1 \end{matrix}\right. \end{aligned}$$

and the dispersion

5 $$\begin{aligned} {\tilde{\sigma }}_{\ln {(C_R)}} = \left\{ \begin{matrix} 0 &{} &{} R \le 1 \\ \left[ \frac{1}{\beta _1} + \frac{1}{\beta _2 \cdot (T+0.1)} \right] \cdot \beta _3 \cdot \left[ 1 - e^{-\beta _4(R-1)} \right] &{} &{} R > 1 \end{matrix}\right. \end{aligned}$$

of the data (these equations are reproduced herein for completeness). In this study, we adopt $\theta _1$ = 79.12 and $\theta _2$ = 1.98 for the counted median in Eq. 4 and $\beta _1 = 5.876$, $\beta _2 = 11.749$, $\beta _3 = 1.957$, and $\beta _4 = 0.739$ in Eq. 5 (Ruiz-García and Miranda 2007).

Ruiz-García and Miranda (2007) also characterized the empirical cumulative probability distribution of $C_R$ using a lognormal probability distribution with the mean of $\ln ({\tilde{C}}_R)$ and the standard deviation equal to ${\tilde{\sigma }}_{\ln {(C_R)}}$. If follows that, given a strength reduction factor *R*, the lognormal cumulative probability distribution of displacement ductility $\mu =C_R R$ can be calculated using Eqs. 4 and 5, as long as the ground motion suite used by Ruiz-García and Miranda (2007) adequately describes the considered seismic hazard.

Finally, observing the graphs in Figs. 1 and 2 of Ruiz-García and Miranda (2007), we note that the geometric mean of $C_R$ is approximately equal to 1 for SDOF systems with vibration periods $T>1.0\textrm{s}$, albeit with a logarithmic standard deviation of between 0.2 and 0.4. Based on this, we assume that the maximum displacements of the inelastic and elastic SDOF oscillators with the same natural vibration periods $T>1.0\textrm{s}$ are statistically the same, and interpret the well-known $R=\mu$ equal displacement rule in this sense.

### The yield point spectrum

The YPS (Aschheim and Black 2000) is a graphical representation of the inelastic response of a SDOF oscillator and the elastic and inelastic seismic demand, in a format similar to the ADRS (acceleration displacement response spectrum) used in the capacity spectrum method (Freeman 1978) and the N2 Method (Fajfar 2000). The main difference between the YPS and the ADRS is that the YPS plots the pseudo-spectral acceleration at yield $S_{a,y}$ versus the yield displacement $S_{d,y}$ for a SDOF oscillator, as shown in Fig. 2a, while the ADRS plots the maximum spectral acceleration versus the maximum spectral displacement ($S_a$ versus  $S_d$ as shown in Fig. 2b) for a SDOF oscillator. In both YPS and ADRS, a relation between the strength reduction factor, the displacement ductility, and the natural vibration period of a SDOF oscillator, the *R*-$\mu$-*T* relation, is used to compute the constant-displacement ductility inelastic seismic design spectra, starting with the elastic seismic design spectrum (Chopra 2016). The capacity curve of the SDOF oscillator is plotted in the corresponding coordinates. This capacity curve is a bilinearization of the principal-mode inelastic static pushover response curve of the structure, converted from the structure to the SDOF oscillator coordinates.

### $\upmu$ -R*-*H/B* relation

The yield displacement of a structure is practically invariant (Priestley 2000; Aschheim 2002) if its geometry and the material properties are maintained in the design process. This is the premise of a family of Constant Yield Displacement (CYD) design procedures, such as the YPS design procedure (Aschheim and Black 2000; Aschheim 2002; Aschheim et al. 2019). However, the YPS design procedure relies on seismic design spectra (Fig. 2a) computed using *R*-$\mu$-*T* relations that are, in turn, derived assuming that the vibration period of the structure remains constant through design iterations (Chopra 2016). To address this inconsistency, Tsiavos and Stojadinović (2019) developed the $\mu$-$R^*$-*H*/*B* relation based on the premise that the yield displacement of the structure remains constant during design iterations, the CYD assumption. The relevant elements of this derivation are repeated here for consistency.

**Fig. 3:**
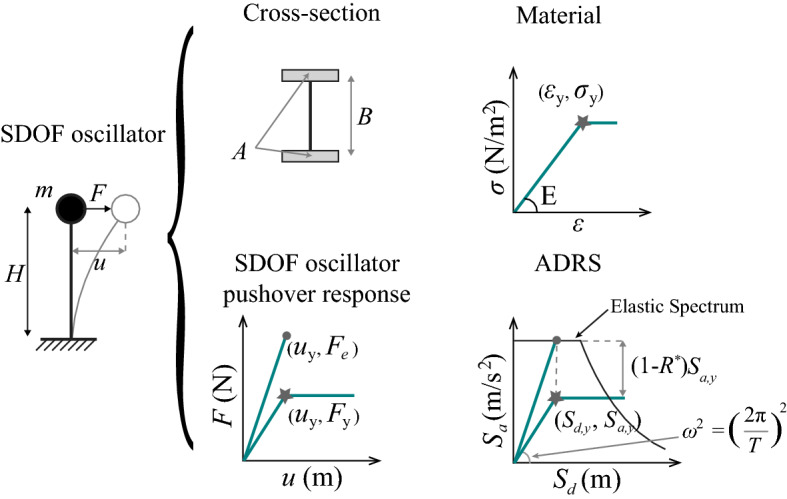
Constant yield displacement (CYD) SDOF oscillator

Tsiavos and Stojadinović (2019) expressed the dynamic response of a prototype structure using a SDOF oscillator surrogate. The SDOF oscillator is excited by a horizontal ground motion applied at its base and responds in pure bending. Thus, it comprises a deformable cantilever beam of height *H* and a concentrated top mass *m* (Fig. 3). The cross section of the SDOF oscillator cantilever column is I-shaped, with two identical symmetrically positioned areas *A* of structural material that responds axially, and a web that provides rigidity in shear. The distance between the material area centroids is *B*. The mechanical properties of the cross-section material are defined by an elastic-perfectly-plastic stress–strain relation, characterized by the material elastic stiffness modulus *E*, yield strain $\varepsilon _y$, and yield stress $\sigma _y=E\varepsilon _y$. Consequently, assuming the cross section remains plane, the cross-section yield curvature $\varphi _y = 2 \varepsilon _y / B$, while the yield displacement of the SDOF oscillator (Fig. 3) is:

6 $$\begin{aligned} u_y = \frac{2}{3} \varepsilon _y H \left( \frac{H}{B} \right) \end{aligned}$$

and its yield strength is:

7 $$\begin{aligned} F_y = E A \varepsilon _y \left( \frac{B}{H} \right) \end{aligned}$$

Assuming that the structural material of the SDOF oscillator remains unchanged, its yield strength can be varied by changing the areas *A* of the structural material without changing the distance between the centroids *B*. Notably, the yield displacement of the SDOF oscillator remains constant as its yield strength varies, because it depends only on the geometry of the oscillator (its height *H* and aspect ratio *H*/*B*) and the yield strain of its structural material $\varepsilon _y$.

The relations between the dynamic characteristics of the prototype structure and its SDOF oscillator surrogate are established using the equivalence of the base shear forces to compute the SDOF oscillator mass *m*, and the equivalence of the base overturning moment to compute the SDOF oscillator height *H*. Also notable is the role of the SDOF oscillator aspect ratio *H*/*B*: it represents the kinematics of the yield mechanism of the prototype structure.

According to Tsiavos and Stojadinović (2019), the strength reduction factor $R^*$ is the ratio of the strength that is required to maintain the response of the SDOF oscillator in the elastic range $F_e = m \cdot S_{a}$ to the yield strength $F_y = m \cdot S_{a,y}$ of the SDOF oscillator based on the premise that its yield displacement remains constant, as shown in Fig. 3.

### Characteristic length ratio

For the purpose of dimensional analysis, we select two lengths, one that characterizes the design-basis seismic hazard and another one that characterizes the SDOF oscillator size. We choose the product $S_s T_c^2$ to be the characteristic length of the design-basis seismic hazard, similar to the pulse length $a_p \cdot T_p^2$ adopted by Makris and Black (2004). This quantity has units of length and is derived directly from the spectral values $S_s$ and $S_1$ that define the elastic design spectra in modern seismic design codes. To characterize the SDOF oscillator size *H*, we use Eq. 6 to derive two length quantities: $u_y/(H/B)=(2/3)\varepsilon _y H$. Finally, the ratio of the SDOF oscillator size and design-basis seismic hazard characteristic length $\kappa$ is defined as:

8 $$\begin{aligned} \kappa = \frac{S_{d,y}}{S_{s} T_c^2 (H/B)} = \frac{ (2/3) \varepsilon _y H }{S_{s} T_c^2} \end{aligned}$$

This ratio represents the yield displacement of the SDOF oscillator normalized by its aspect ratio and the seismic hazard characteristic length. Importantly, it enables expressing the ductility-strength and the strength-ductility relations in a dimensionless way, as a function of the aspect ratio of the SDOF oscillator, following the rationale in Tsiavos and Stojadinović (2019).

## Derivation of the ductility-strength relations in the CYD framework

Starting with a simplified elastic design spectrum that expresses the design-basis seismic hazard, we aim to derive a relation between the displacement ductility and the strength of a SDOF oscillator whose response is inelastic in design-basis hazard events. The yield displacement of this SDOF oscillator $u_y = S_{d,y}$, its yield strength $F_y = m S_{a,y}$ and its force-displacement response envelope is elastic-perfectly-plastic, as shown in Fig. 3.

**Fig. 4:**
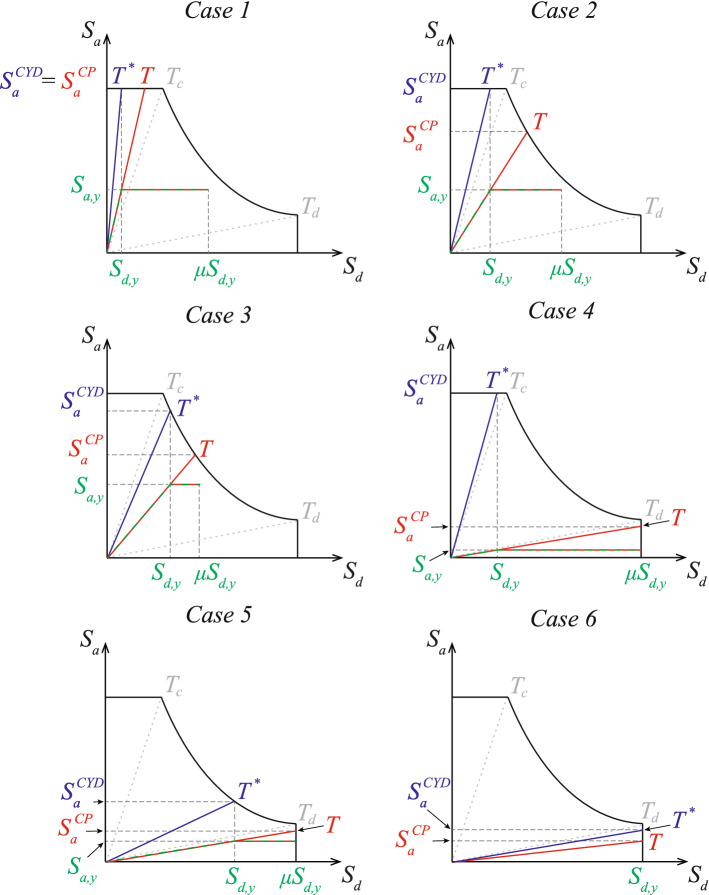
The yielding SDOF oscillator and its elastic counterparts, the CP-SDOF and CYD-SDOF oscillators, shown in the ADRS format for the six considered cases

We construct two linear-elastic counterparts for this inelastic SDOF oscillator, as shown in Fig. 4 using the ADRS format. The elastic counterpart of the SDOF oscillator whose yield displacement remains constant, the CYD-SDOF elastic oscillator, has a (variable) vibration period $T^*=2\pi \sqrt{S_{d,y}/S_{a}^{CYD}}$, where $S_{a}^{CYD} = F_e^{CYD}/m$ corresponds to the strength required to maintain the response in the elastic range for the design-basis seismic hazard. The SDOF elastic oscillator whose period remains constant, the CP-SDOF elastic oscillator, has a vibration period $T=2\pi \sqrt{S_{d,y}/S_{a,y}}$. Analogously, $S_{a}^{CP} = F_e^{CP}/m$ corresponds to the minimum strength of the CP-SDOF oscillator required to maintain its response in the elastic range for the design-basis seismic hazard. Comparing the CYD-SDOF and the CP-SDOF elastic surrogate oscillators across a range of possible yield displacements, it is apparent that $T^* \le T$. Similarly, given that the SDOF oscillator is designed to yield, $S_{a}^{CYD} \ge S_{a}^{CP} > S_{a,y}$.

Six different cases delineate the behavior of the inelastic SDOF oscillator. They are distinguished by locating the vibration periods of the CYD-SDOF $(T^*)$ and CP-SDOF (*T*) elastic SDOF oscillators in different regions of the simplified elastic design spectrum, as shown in Fig. 4. Next, we derive the $\mu$-$R^*$-$S_{d,y}$ ductility-strength relation from the $C_R$-*R*-*T* relation (Ruiz-García and Miranda 2007) using the definitions of the strength reduction factors $R^*$ and *R* in each case. In parallel, we derive the $\mu$-$R^*$-*H*/*B*-$\kappa$ relation to use in dimensional analysis. Finally, we address the case when the response of the SDOF oscillator remains elastic in design-basis hazard events.

### Case 1

In this case, the period of the CP-SDOF oscillator is in the constant acceleration region of the elastic design spectrum ($T \le T_c$, Fig. 4 top row, left). The CYD-SDOF oscillator is in the same spectral region, since $T^*\le T$. Thus, the strength values that are required to maintain the response of both oscillators in the elastic range under the design-basis hazard are equal, leading to $S_{s} = S_{a}^{CYD}= S_{a}^{CP}$. Therefore, the CP and CYD strength reduction factors are equal:

9 $$\begin{aligned} R = R^* = \frac{S_{s}}{S_{a,y}} \end{aligned}$$

The threshold between Case 1 and Case 2 is reached when CP-SDOF oscillator is at the end of the constant acceleration region of the elastic design spectrum, i.e., $T = T_c$. We restate this threshold in terms of the yield displacement of the SDOF oscillator using Eq. 2:

10 $$\begin{aligned} S_{d,y1} = \left( \frac{T_c}{2\pi } \right) ^2 S_{a,y} = \frac{T_c^2 S_{s}}{4 \pi ^2 R} = \frac{T_c^2 S_{s}}{4 \pi ^2 R^*} \end{aligned}$$

To relate the displacement ductility $\mu$ of the SDOF oscillator and its strength reduction factor $R^*$ we rewrite Eq. 4, given that $C_R = \mu / R$, as a function of $R^*$, $S_{d,y}$, and $S_{s}$:

11 $$\begin{aligned} \mu = R^* \left( 1 + \left( \frac{1}{\theta _1 \left( 2 \pi \sqrt{\frac{S_{d,y}R^*}{S_{s}} } \right) ^{\theta _2}} \right) (R^*-1) \right) , \; \text {for} \; S_{d,y} \le S_{d,y1} \end{aligned}$$

where parameters $\theta _1$ and $\theta _2$ are taken from Ruiz-García and Miranda (2007).

Simultaneously, using Eq. 8, we express the threshold $S_{d,y1}$ between Case 1 and Case 2 in terms of the characteristic length ratio $\kappa _1$:

12 $$\begin{aligned} \kappa _1 = \frac{1}{4 \pi ^2 R^* (H/B)} \end{aligned}$$

Finally, we restate Eq. 11 using the characteristic length ratio $\kappa$ (Eq. 8) as:

13 $$\begin{aligned} \mu = R^* \left( 1 + \left( \frac{1}{\theta _1 \left( 2 \pi \sqrt{ R^* \kappa T_c^2 (H/B) } \right) ^{\theta _2}} \right) (R^*-1) \right) , \; \text {for} \; \kappa \le \kappa _1 \end{aligned}$$

### Case 2

In this case, the period of the CP-SDOF oscillator is in the constant velocity region ($T_c < T \le T_d$), while the period of the CYD-SDOF oscillator remains in the constant acceleration region of the elastic design spectrum ($T^* \le T_c$). The graph on the right of the top row of Fig. 4 for Case 2 indicates that: (i) $S_{a}^{CP}=S_1/T$, (ii) $S_{a}^{CYD}=S_{s}$. Thus, the relation between the CP and the CYD strength reduction factors is:

14 $$\begin{aligned} R = \frac{S_{a}^{CP}}{S_{a,y}}= \frac{S_{s}T_c}{T} \frac{1}{S_{a,y}} = \frac{S_{s}T_c \sqrt{S_{a,y}}}{2 \pi \sqrt{S_{d,y}}} \frac{1}{S_{a,y}} = \frac{S_{s}T_c}{2\pi \sqrt{S_{a,y} S_{d,y}}} = \frac{T_c \sqrt{S_{s} R^*}}{2\pi \sqrt{S_{d,y}}} \end{aligned}$$

The period of the CYD-SDOF oscillator $T^*=T_c$ at the threshold between Case 2 and Case 3. Applying Eq. 2, we state this threshold as a function of the SDOF oscillator yield displacement:

15 $$\begin{aligned} S_{d,y2} = \frac{T_c^2 S_{s}}{4\pi ^2} \end{aligned}$$

Inserting Eqs. 2 and 14 into Eq. 4, SDOF oscillator displacement ductility $\mu$ can be written as a function of $R^*$, $S_{d,y}$, $S_{s}$ and $T_c$:

16 $$\begin{aligned} \mu = \frac{T_c \sqrt{S_{s} R^*}}{2\pi \sqrt{S_{d,y}}} \left( 1 + \left( \frac{1}{\theta _1 \left( 2 \pi \sqrt{\frac{S_{d,y}R^*}{S_{s}}} \right) ^{\theta _2} } \right) \left( \frac{T_c \sqrt{S_{s} R^*}}{2\pi \sqrt{S_{d,y}}} - 1 \right) \right) , \; \text {for} \; S_{d,y1} < S_{d,y} \le S_{d,y2} \end{aligned}$$

Again, using the characteristic length ratio $\kappa$ from Eq. 8, the threshold between Case 2 and Case 3 is restated:

17 $$\begin{aligned} \kappa _{2} = \frac{1}{4 \pi ^2 (H/B)} \end{aligned}$$

Finally, the $\mu$-$R^*$-*H*/*B*-$\kappa$ relation in Case 2 is:

18 $$\begin{aligned} \mu = \frac{\sqrt{R^*}}{2\pi \sqrt{\kappa (H/B)}} \left( 1 + \left( \frac{1}{\theta _1 \left( 2 \pi \sqrt{\kappa T_c^2 (H/B) R^*} \right) ^{\theta _2} } \right) \left( \frac{\sqrt{R^*}}{2\pi \sqrt{\kappa (H/B)}} - 1 \right) \right) , \; \text {for} \; \kappa _1 < \kappa \le \kappa _{2} \end{aligned}$$

### Case 3

The CYD-SDOF oscillator joins the CP-SDOF oscillator in the constant velocity region of the elastic design spectrum in Case 3, meaning that $T_c < T^* \le T \le T_d$ (Fig. 4 middle row, left). Thus: (i) $S_{a}^{CP}= S_1/T$, (ii) $S_{a}^{CYD}=S_1/T^*$. Then, the relationship between *R* and $R^*$ is, as in Tsiavos and Stojadinović (2019):

19 $$\begin{aligned} R = \sqrt{R^*} \end{aligned}$$

The threshold between Cases 3 and 5 is attained when the CP-SDOF oscillator reaches the end of the constant velocity region of the elastic design spectrum, i.e., $T=T_d$. The displacement of the CP-SDOF elastic oscillator is then equal to the maximum elastic spectral displacement:

20 $$\begin{aligned} S_{d,max} = \frac{S_{s} T_c T_d}{4\pi ^2} \end{aligned}$$

Using Eq. 2 and the equal displacement rule adopted in Sect. 2.2, we state the yield displacement threshold as a function of the SDOF oscillator yield displacement:

21 $$\begin{aligned} S_{d,y3} = \frac{S_{d,max}}{\mu } = \frac{S_{d,max}}{R} = \frac{S_{d,max}}{\sqrt{R^*}} = \frac{T_c T_d S_{s}}{4 \pi ^2 \sqrt{R^*}} \end{aligned}$$

Applying Eqs. 6 and 8, we restate the threshold in Eq. 21 in terms of the characteristic length ratio:

22 $$\begin{aligned} \kappa _3 = \frac{1}{4 \pi ^2 (H/B)} \frac{T_d}{\mu T_c} = \frac{T_d}{4 \pi ^2 (H/B) T_c \sqrt{R^*}} \end{aligned}$$

Substituting Eq. 19 into Eq. 4, we rewrite the SDOF oscillator displacement ductility $\mu$ as a function of $R^*$, $S_{d,y}$, $S_{s}$, and $T_c$:

23 $$\begin{aligned} \mu = \sqrt{R^*} \left( 1+ \left( \frac{1}{\theta _1 \left( \frac{4\pi ^2 S_{d,y}\sqrt{R^*}}{S_{s}T_c} \right) ^{\theta _2}} \right) \left( \sqrt{R^*} - 1 \right) \right) , \; \text {for} \; S_{d,y2}< S_{d,y} < S_{d,y3} \end{aligned}$$

and, finally, obtain the $\mu$-$R^*$-*H*/*B*-$\kappa$ relation:

24 $$\begin{aligned} \mu = \sqrt{R^*} \left( 1+ \left( \frac{1}{\theta _1 \left( 4\pi ^2 T_c \kappa (H/B) \sqrt{R^*} \right) ^{\theta _2}} \right) \left( \sqrt{R^*} - 1 \right) \right) , \; \text {for} \; \kappa _2< \kappa < \kappa _3 \end{aligned}$$

### Cases 4, 5 and 6

Cases 4, 5 and 6 are shown in the middle and bottom rows of Fig. 4. Common to all three cases is that the period of the CP-SDOF oscillator $T \ge T_d$, putting the CP-SDOF oscillator in the constant displacement region of the elastic design spectrum. Then, the strength of the CP-SDOF elastic oscillator $S_{a}^{CP}= T_c T_d S_s/T^2$ varies with its period *T*, but its displacement $S_{d}^{CP}$ equals the maximum elastic spectral displacement $S_{d,max}$ (Eq. 20) regardless of its period. Applying Eq. 6 and 8, the limit given by Eq. 20 as a function of the characteristic length ratio is:

25 $$\begin{aligned} \kappa _{max} = \frac{1}{4 \pi ^2 (H/B)} \frac{T_d}{T_c} \end{aligned}$$

The yield displacement of the SDOF oscillator $S_{d,y} = S_{d,y3} < S_{d,y2}$ in Case 4 and $S_{d,y} = S_{d,y3} > S_{d,y2}$ in Case 5. Thus, the CYD-SDOF elastic oscillator is in the constant acceleration and constant velocity regions of the elastic design spectrum, respectively. We consider that the equal displacement rule adopted in Sect. 2.2 is valid in all three cases, as $T> T_d > 1\textrm{s}$ almost always. Therefore, the displacement ductility of the SDOF oscillator in all three cases is:

26 $$\begin{aligned} \mu = \frac{S_{d,max}}{S_{d,y}} = \frac{T_c T_d S_s}{4 \pi ^2 S_{d,y}}, \; \text {for} \; S_{d,y} = S_{d,y3} \le S_{d,max} \end{aligned}$$

or, equivalently:

27 $$\begin{aligned} \mu = \frac{T_d}{4 \pi ^2 \kappa T_c (H/B)}, \; \text {for} \; \kappa = \kappa _3 \le \kappa _{max} \end{aligned}$$

It is worth noting the displacement ductility value that sets the boundary between Case 4 and Case 5 is a function of the elastic design spectrum corner periods. Namely, Case 4 can happen if $\mu > T_d / T_c$, as $S_{d,y2} > S_{d,y3}$, while Case 5 can happen if $\mu < T_d / T_c$ as $S_{d,y2} < S_{d,y3}$.

**Fig. 5:**
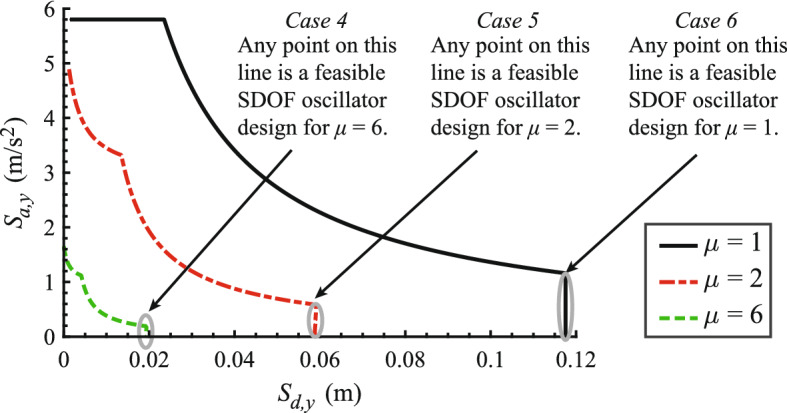
Illustration of the infinite number of possible SDOF oscillators with a given yield displacement $S_{d,y}$ in Cases 4, 5 and 6

Remarkably, the SDOF oscillator displacement ductility depends only on its yield displacement (or its aspect ratio), but not on its yield strength, as long as its vibration period *T* is greater than $T_d$, therefore satisfying Cases 4, 5 and 6 condition that the CP-SDOF oscillator is in the constant displacement region of the elastic design spectrum. In turn, this means that infinitely many SDOF oscillators with the same yield displacement and different yield strengths develop the same displacement ductility under the design-basis hazard, as shown in Fig. 5.

The relation between the CP strength reduction factor *R* and the SDOF oscillator displacement ductility $\mu$ is governed by the equal displacement rule: $R=\mu$ regardless of the SDOF oscillator yield strength. However, the CYD strength reduction factor $R^*$ of the SDOF oscillator depends on its yield strength. Thus, in the CYD framework, there are infinitely many $R^*$ values associated with a SDOF oscillator whose displacement ductility is given by Eq. 26 (Cases 4, 5 and 6). To resolve this issue, we select a unique SDOF oscillator as the one that has the largest yield strength (and, therefore, the largest stiffness, and the smallest CYD strength reduction factor $R_{min}^*$) for a given yield displacement. This criterion corresponds exactly to the CP-SDOF oscillator with vibration period $T=T_d$. The CYD strength reduction factor of this SDOF oscillator is:

28 $$\begin{aligned} R^*_{min} = {\left\{ \begin{array}{ll} {\mu T_d}/{T_c} &{} \; \text {for} \; \text {Case 4} \; : \; S_{d,y} = S_{d,y3}< S_{d,y2}\\ \mu ^2 &{} \; \text {for}\; \text {Case 5} \; : \; S_{d,y2}< S_{d,y} = S_{d,y3} < S_{d,max} \\ 1 &{} \; \text {for} \; \text {Case 6} \; : \; S_{d,y}= S_{d,y3} = S_{d,max} \end{array}\right. } \end{aligned}$$

### Elastic SDOF oscillator

It is possible that the structure, and therefore its SDOF oscillator surrogate, does not yield under the considered seismic hazard. In the design process, this happens if the SDOF oscillator has a yield displacement $S_{d,y} \ge S_{d,max}$ (equivalently, the characteristic length ratio $\kappa \ge \kappa _{max}$). Therefore, it remains elastic under the design seismic hazard regardless of its strength. Such a situation may occur in regions of low seismic hazard, or when the structure represented by the SDOF oscillator surrogate is deformable and/or slender. Consequently, other prescribed loads will govern the design.

In the evaluation process, the elastic SDOF oscillator surrogate is identified when $R^* \le 1$. This situation is covered in Ruiz-García and Miranda (2007): if $R \le 1$ then $C_R = 1$. In the CYD framework, seismic evaluation of an elastic SDOF oscillator is equally straightforward: if $S_{d,y} \le S_{d,y1}$, then $\mu = S_s/S_{a,y}$ and if $S_{d,y} > S_{d,y1}$ then $\mu = R = \sqrt{R^*}$.

### Ductility-strength relations graph

**Fig. 6:**
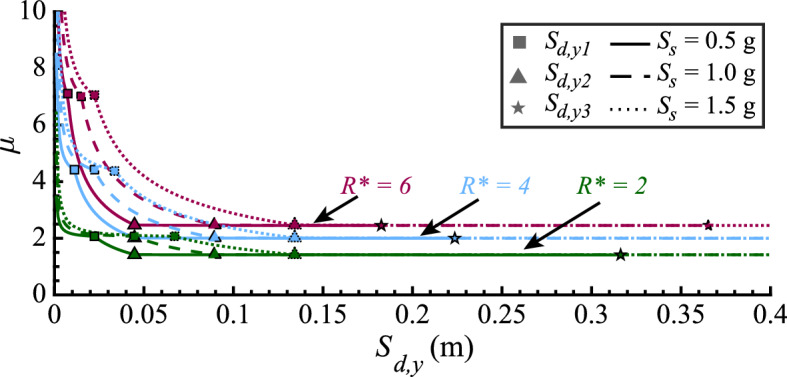
Graphs of the $\mu$-$R^*$-$S_{d,y}$ relation for three different seismic behavior categories represented by $R^*={2,4,6}$ and three different design-basis seismic hazard intensities, represented by $S_s =$ 0.5 g, 1.0 g, 1.5 g, $T_c = 0.6s$ and $T_d = 6s$. Thresholds $S_{d,y1}$, $S_{d,y2}$ and $S_{d,y3}$ are also shown

The $\mu$-$R^*$-$S_{d,y}$ relation derived above is shown in Fig. 6. The graphs are computed for three different values of the CYD strength reduction factor $R^*$, representing different seismic behavior categories of the prototype structure, and three different elastic spectral acceleration values $S_s$, representing different design-basis seismic hazards. The elastic design spectra are defined by setting $T_c = 0.6s$ (consequently, $S_1 = S_s T_c$) and $T_d = 6s$. Thus, the spectral displacement $S_{d,max}$ values of 0.45 m, 0.89 m and 1.34 m correspond to the spectral acceleration $S_s$ values of 0.5 g, 1.0 g and 1.5 g.

The $\mu$-$R^*$-$S_{d,y}$ graphs shown in Fig. 6 reflect Case 1, 2 and 3 relations between SDOF oscillator displacement ductility and yield displacement, parameterized by its CYD strength reduction factor $R^*$. Note that the case threshold $S_{d,y2}$ values are the same for the same value of spectral acceleration $S_s$. The minimum strength reduction factor $R_{min}^*$ and displacement ductility $\mu$ pairs derived in Cases 4, 5 and 6 are not shown for clarity, as they occur when $S_{d,y} = S_{d,y3}$. We provide a spreadsheet (Silva et al. 2022) to calculate Eqs. 11, 16, and 23 that define the $\mu$-$R^*$-$S_{d,y}$ relation shown in Fig. 6.

## Derivation of the strength-ductility relations in the CYD framework

The $\mu$-$R^*$-$S_{d,y}$ and $\mu$-$R^*$-*H*/*B*-$\kappa$ relations between the displacement ductility and the yield strength of a SDOF oscillator, derived in the previous section, are the basis for deriving the $R^*$-$\mu$-$S_{d,y}$ and $R^*$-$\mu$-*H*/*B*-$\kappa$ relations between the strength reduction factor and the displacement ductility of the same SDOF oscillator. We perform this inversion exactly, using a numerical approach, and approximately, introducing simplifications.

First, we round the value of parameter $\theta _2$ in the $C_R$-*R*-*T* relations (Eqs. 4 and 5) from 1.98 to 2.0, to be able to work with square roots. This rounding makes it possible to invert the $\mu$-$R^*$-$S_{d,y}$ relation in closed form. In Case 1, with $\theta _2 = 2$, CYD strength reduction factor is:

29 $$\begin{aligned} R^* = \frac{\mu + S_{s}/(\theta _1 4 \pi ^2 S_{d,y})}{1 + S_{s}/(\theta _1 4 \pi ^2 S_{d,y})} \end{aligned}$$

or as a function of *H*/*B* and $\kappa$:

30 $$\begin{aligned} R^* = \frac{\mu (\theta _1 4 \pi ^2 T_c^2 \kappa (H/B)) + 1}{(\theta _1 4 \pi ^2 T_c^2 \kappa (H/B)) + 1} \end{aligned}$$

The threshold between Case 1 and Case 2 is derived by inserting Eq. 29 into Eq. 10 (respectively Eq. 12 for $\kappa _1$ threshold).

In Case 2, Eq. 16 is first rewritten considering $\theta _2 = 2$ as follows:

31 $$\begin{aligned} \mu = \left( \frac{T_c \sqrt{S_{s}}}{2\pi \sqrt{S_{d,y}}} \right) \sqrt{R^*} - \left( \frac{T_c S_{s}^{1.5}}{8\pi ^3 \theta _1 S_{d,y}^{1.5}} \right) \frac{1}{\sqrt{R^*}} + \frac{T_c^2 S_{s}^2}{16\pi ^4 \theta _1 S_{d,y}^2} \end{aligned}$$

This equation is inverted to obtain:

32 $$\begin{aligned} R^* = \frac{S_{s}}{4 \pi ^2 S_{d,y} \theta _1} + \frac{2 \pi ^2 S_{d,y} C_1^2}{S_{s} T_c^2} + \frac{2 \pi ^2 S_{d,y} C_1 }{S_{s} T_c^2} \sqrt{C_1^2 + \frac{T_c^2 S_{s}^2}{4 \pi ^4 S_{d,y}^2 \theta _1}} \end{aligned}$$

where $C_1$ is defined as:

33 $$\begin{aligned} C_1 = \frac{-T_c^2 S_{s}^2}{16 \pi ^4 S_{d,y}^2 \theta _1} + \mu \end{aligned}$$

Note that the value of $\mu$ is larger than 1, and usually much larger than $(T_c^2 S_{s}^2)/(16 \pi ^4 S_{d,y}^2 \theta _1)$. Therefore we may consider $C_1 \approx \mu$. Equations 32 and 33 rewritten as functions of *H*/*B* and $\kappa$ are:

34 $$\begin{aligned} R^* = \frac{1}{4 \pi ^2 \theta _1 T_c^2 \kappa (H/B)} + 2 \pi ^2 \kappa (H/B) C_1^2 + 2 \pi ^2 \kappa (H/B) C_1 \sqrt{C_1^2 + \frac{1}{4 \pi ^4 \theta _1 T_c^2 \kappa ^2 (H/B)^2}} \end{aligned}$$

and

35 $$\begin{aligned} C_1 = \frac{-1}{16 \pi ^4 \theta _1 T_c^2 \kappa ^2 (H/B)^2} + \mu \end{aligned}$$

The threshold between Case 2 and Case 3 given by Eq. 15 is a constant and does not need to be modified. For Case 3, Eq. 23 rewritten considering $\theta _2 = 2$ is:

36 $$\begin{aligned} \mu = \sqrt{R^*} - \left( \frac{T_c^2 S_{s}^{2}}{16\pi ^4 \theta _1 S_{d,y}^{2}} \right) \frac{1}{\sqrt{R^*}} + \frac{T_c^2 S_{s}^2}{16\pi ^4 \theta _1 S_{d,y}^2} \end{aligned}$$

and symbolically inverted as follows:

37 $$\begin{aligned} R^* = C_2 - C_2 \mu +\frac{{C_2 }^2 }{2}-\frac{{\left( C_2 -\mu \right) } \sqrt{{C_2 }^2 -2 C_2 \mu +4 C_2 +\mu ^2 }}{2}+\frac{\mu ^2 }{2} \end{aligned}$$

where $C_2$ is defined as:

38 $$\begin{aligned} C_2 = \frac{T_c^2 S_{s}^{2}}{16\pi ^4 \theta _1 S_{d,y}^{2}} \end{aligned}$$

Note that the value of $(T_c^2 S_{s}^2)/(16 \pi ^4 S_{d,y}^2 \theta _1)$ is very small. Therefore, we may take $C_2 \approx 0$ and $\mu \approx \sqrt{R^*}$, which is equivalent to considering that the equal displacement rule is valid for the entire Case 3. Yet, if one wants to compute the exact solution, then Eq. 38 is also presented as a function of *H*/*B* and $\kappa$:

39 $$\begin{aligned} C_2 = \frac{1}{16\pi ^4 \theta _1 T_c^2 \kappa ^2 (H/B)} \end{aligned}$$

The threshold between Case 3 and Case 5, as well as the threshold between Case 2 and Case 4, are derived by inserting Eq. 37 into Eq. 21 to compute $S_{d,y3}$, or into Eq. 22. Lastly, in Cases 4, 5 and 6, the relation between the minimum CYD strength reduction factor and displacement ductility stated in Eq. 28 is adopted without modifications.

**Fig. 7:**
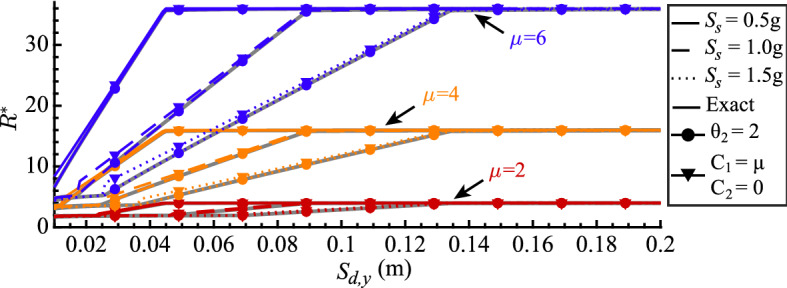
Graphs of the $R^*$-$\mu$-$S_{d,y}$ relations for three different seismic behavior categories represented by $\mu ={2,4,6}$ and three different design-basis seismic hazard intensities, represented by $S_s={0.5g, 1.0g, 1.5g}$ and $T_c = 0.6\,s$. Each relation is computed in three ways: exactly (Eqs. 11, 16 and 23), assuming $\theta _2 = 2.0$ (Eqs. 29, 32, and 37), and further simplifying the latter equations by setting $C_1 = \mu$ and $C_2 = 0$

The exact, numerically inverted, and the approximate, symbolically inverted, $R^*$-$\mu$-$S_{d,y}$ relations are plotted in Fig. 7. They are computed for three different values of the SDOF oscillator displacement ductility $\mu$, representing different seismic behavior categories of the structure, and three different spectral acceleration values $S_s$, representing different design-basis seismic hazards. Evidently, rounding $\theta _2$ to 2.0 introduces a small error compared to the exact, numerically inverted, $R^*$-$\mu$-$S_{d,y}$ relation computed with $\theta _2 = 1.98$. However, simplifying Eqs. 29, 32, and 37 further by taking $C_1 = \mu$ and $C_2 = 0$ introduces small but appreciable errors, particularly in Case 2 for fairly high displacement ductility values (e.g., $\mu =6$ in Fig. 7).

Based on this comparison, we provide two options for using $R^*$-$\mu$-$S_{d,y}$ relations in practice: (i) adopt Eqs. 29, 32, and 37 simplified by setting $C_1 = \mu$ and $C_2 = 0$, or (ii) use a spreadsheet (Silva et al. 2022) to perform the required calculations using Eqs. 29, 32, and 37 with $\theta _2 = 2.0$.

## Seismic design in the CYD framework

The $R^*$-$\mu$-$S_{d,y}$ strength-ductility relations derived in the previous section are the basis for risk-informed, deterministic, displacement-based seismic design of structures. These strength-ductility relations are used to design a new structure with respect to the design-basis seismic hazard as follows:

- Preliminary steps:
  - Specify the seismic hazard values of $S_s$, $S_1$, and $T_d$ to define the design-basis elastic seismic design spectrum. If $T_d$ is not available, select a value several times larger than $T_c = S_1/S_s$.
  - Specify the geometry and mass distribution of the new structure and the yield strain of the yielding material.
  - Select the intended seismic behavior category of the new structure and specify its deformation ductility capacity.
- Step 1: Compute the yield displacement of the SDOF oscillator surrogate of the new structure $S_{d,y}$.
- Step 2: Determine the case thresholds $S_{d,y1}$, $S_{d,y2}$ and $S_{d,y3}$, as well as $S_{d,max}$ using Eqs. 10 (with 29), 15, 21 and 20, respectively.
- Step 3: Calculate SDOF oscillator CYD strength reduction factor $R^*$ corresponding to the design-basis seismic hazard:
  - Case 1 ($S_{d,y} \le S_{d,y1}$): calculate $R^*$ using Eq. 29.
  - Case 2 ($S_{d,y1} < S_{d,y} \le S_{d,y2}$): calculate $R^*$ using Eq. 32.
  - Case 3 ($S_{d,y2}< S_{d,y} < S_{d,y3}$): calculate $R^*$ using Equation  37.
  - Cases 4, 5 and 6 ($S_{d,y} \ge S_{d,y3}$): if $S_{d,y} > S_{d,y3}$ back-calculate $\mu$ using Eq. 26. Calculate $R^*_{min}$ using Eq. 28.
  - Elastic behavior ($S_{d,y} > S_{d,y,max}$): the SDOF oscillator surrogate of the new structure remains elastic under the design-basis seismic hazard.
- Step 4: Compute the the yield spectral acceleration $S_{a,y}$ for the SDOF oscillator surrogate of the new structure:
  - Case 1 and 2: $$S_{a,y} = S_{s}/R^*$$.
  - Case 3: $$S_{a,y} = (S_{s}T_c)^2/(4 \pi ^2 S_{d,y} R^*)$$.
  - Cases 4, 5 and 6: $$S_{{{a,y}_{max}}} = S_{s} T_c / (T_d \mu )$$.
- Step 5: Compute the seismic forces in the new structure and size its elements for the design-basis hazard.
- Repeat Step 1 to Step 5 until the yield displacement $S_{d,y}$ of the SDOF oscillator surrogate of the new structure converges. If the yield strain and the geometry (size and aspect ratio) of the SDOF oscillator surrogate of the new structure remain the same while its yield strength changes, this design iteration should converge in a few steps.

The presented CYD procedure for seismic design of a new structure is exemplified in the following sections. The CYD seismic design procedure is similar to the YPS design procedure (Aschheim and Black 2000; Aschheim et al. 2019). Both procedures are displacement-based and utilize the CYD assumption to minimize the number of design iterations. However, the YPS design procedure requires drawing a graph that comprises a wide range of periods and yield displacements using a *R*-$\mu$-*T* relation. In contrast, the CYD design procedure uses the derived $R^*$-$\mu$-$S_{d,y}$ relation and, thus, requires neither the information about the period of the designed structure, nor drawing a graph. Notably, the CYD seismic design procedure differs from CP seismic design procedures (e.g, Example 7.3 in Chopra, 2016) because the yield displacement is controlled by the geometry and mechanical characteristics of the structure and cannot attain unrealistically small values, as pointed out by Vassiliou et al. (2013) and Tsiavos et al. (2017, 2021b).

### Seismic design example

The building examined in Hernández-Montes and Aschheim (2019) is used herein. This is a four-story reinforced concrete moment-resisting frame building located in San Jose California (site class D, $S_{MS} = S_{s} = 1.0\hbox {g}$, $S_{M1} = S_1 = 0.6\hbox {g}, T_s = T_c = 0.6\hbox {s}, T_L = T_d = 12\hbox {s}$). The objective is to determine the base shear strength of this building given a target deformation ductility $\mu = 2.4$. The yield displacement of the building $u_y \approx 0.09\,m$ and the participation factor $\Gamma \approx 1.29$. Instead of constructing the elastic design spectrum and using the $C_R$-*R*-*T* relationship to construct the yield point spectra, the CYD framework described in this paper is applied as follows:

- Step 1: the yield displacement of the SDOF oscillator surrogate $S_{d,y} = u_y/ \Gamma \approx 0.09/1.29 = 0.07m$.
- Step 2: the yield displacement limits are $S_{d,y1} = 0.038\,m$, $S_{d,y2} = 0.089\,m$, $S_{d,y3} = 0.74\,m$ and $S_{d,y,max} = 1.79\,m$.
- Step 3: as the SDOF oscillator is in Case 2 ($S_{d,y1} \le S_{d,y} < S_{d,y2}$), using Eq. 32, $R^* = 4.36$ (calculated using $\theta _1 = 79.12$).
- Step 4: for Case 2, the yield spectral acceleration is $S_{a,y} = S_{s}/R^* = 0.23g$.

These steps are already automated in the spreadsheet provided in Silva et al. (2022).

The calculated $S_{a,y}$ is the same as the one determined by Hernández-Montes and Aschheim (2019) using the YPS method (therein, $S_{a,y}$ is the base shear force coefficient $C_y^*$).

### Performance-based seismic design example

The reinforced concrete shear wall building described in Tjhin et al. (2007) is adopted for this example. This building has 6 stories and is 22 m tall. The participation factor $\Gamma \approx 1.41$ and the yield displacement $u_y \approx 0.0902\,m$, therefore the yield displacement of the SDOF oscillator $S_{d,y} = u_y / \Gamma \approx 0.0902 / 1.41 = 0.064\,m$. The performance objectives, based on FEMA (2000) and used by Tjhin et al. (2007) are reproduced in Table 1. In this example, the performance objectives stated in terms of the the building displacement ductility demand and the associated seismic hazard intensity (i.e. return period) are used.

**Table 1:** Performance objectives used to design the reinforced concrete shear wall building

Performance objective	Return period	Roof drift (% of building height) Resultant ductility demand	Plastic hinge rotation (rad) Resultant ductility demand
Immediate occupancy	73 years	0.5% 1.22	0.002 rad 1.23
Life Safety	475 years	1.0% 2.43	0.004 rad 1.69
Collapse Prevention	975 years	2.0% 4.87	0.008 rad 2.59

The seismic hazard is defined as in the previous examples, using a site located in San Jose California (site class D, $S_{MS} = S_{s} = 1.0\hbox {g}, S_{M1} = S_1 = 0.6\hbox {g}, T_s = T_c = 0.6\hbox {s}, T_L = T_d = 12\hbox {s}$). This seismic hazard is associated with the 475-year return period, and is taken as the design-basis seismic hazard. The procedure described in Section 1.6.1.3 of FEMA (2000) is used to obtain the seismic hazard for other return periods associated with the performance objectives in Table 1, described using $S_s$, $S_1$ and $T_c$ values. The value of $T_d$ is 12 s and does not vary with the return period.

Then, the CYD framework seismic design procedure described in the paper is performed for each of the three performance objectives, resulting in three CYD strength reduction factors $R^*$ that satisfy the specified displacement ductility demands. Finally, the yield spectral acceleration $S_{a,y}$ values are computed for each performance objective, considering the appropriate case of the CYD design framework. The results are presented in Table 2.

**Table 2:** Input parameters and results of the performance-based seismic design procedure

Performance objective	$S_s$	$T_c$	$\mu$	$S_{d,y1}$	$S_{d,y2}$	$S_{d,y3}$	$S_{d,y,max}$	Case	$R^*$	$S_{a,y}$
Immediate occupancy	0.44 g	0.6 s	1.22	0.0323	0.0392	0.6433	0.7848	3	1.48	0.18 g
Life safety	1.0 g	0.6 s	1.69	0.0540	0.0895	1.0587	1.7891	2	1.98	0.51 g
Collapse prevention	1.23 g	0.6 s	2.59	0.0441	0.1102	0.8510	2.2040	2	3.71	0.33 g

Performance objective Life Safety governs the design, as it requires the largest yield spectral acceleration $S_{a,y}$. Note that $S_{a,y}$ depends not only on $R^*$ but also on the elastic seismic design spectrum associated with the seismic hazard intensity (return period) of each performance objective.

## Dimensional analysis of the CYD ductility-strength and strength-ductility relations

**Fig. 8:**
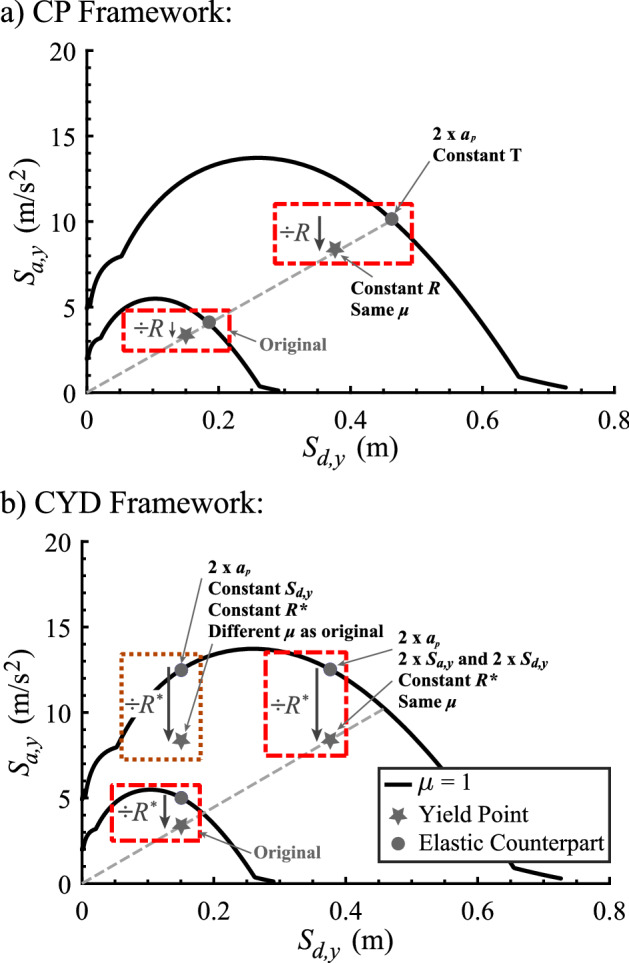
Sketch showing how **a** *R*-$\mu$-*T* relations (CP framework) and **b** $\mu$-$R^*$-$S_{d,y}$ relations (CYD framework) scale with ground motion intensity scaling. The original and amplified ground motion spectra are stylized for clarity. The arrows associated with strength reduction factors *R* and $R^*$ indicate the difference between the yield spectral accelerations of the yielding SDOF oscillator and its elastic CP-SDOF and CYD-SDOF counterparts

The *R*-$\mu$-*T* relation is independent of the design-basis seismic hazard. This is evident from Eq. 3: the resultant displacement ductility $\mu$ is a function of the SDOF oscillator characteristics (its natural frequency $\omega$, damping ratio $\zeta$, and normalized force-displacement function ${\tilde{f}}_s$), its yield spectral acceleration $S_{a,y}$ and the ground motion excitation $\ddot{u}_g$. Two sketches of elastic ground motion response spectra, shown in YPS format in Fig. 8a, are computed using the original and an amplified ground motion acceleration record. Consistent scaling of $\ddot{u}_g$ and $S_{a,y}$ keeps the CP strength reduction factor $R=S_a(T)/S_{a,y}$ constant. Since the SDOF oscillator period $T=2\pi /\omega$ is also kept constant, the resulting ductility $\mu$ is necessarily the same for both the original and the amplified ground motions.

The $\mu$-$R^*$-$S_{d,y}$ relation, on the other hand, depends on the design-basis seismic hazard: SDOF oscillator displacement ductility $\mu$ depends on $S_{s}$ and $T_c$ in Eqs. 11, 16 and 23. The sketch in Fig. 8b shows that amplifying the ground motion, while preserving the SDOF oscillator yield displacement $S_{d,y}$ and its CYD strength reduction factor $R^*$, does not guarantee the same maximum SDOF oscillator displacement ductility $\mu$ as the period of the SDOF oscillator changes.

Inspired by the work of Makris and Black (2004), a dimensional analysis of the equation of motion of the elastic-perfectly-plastic SDOF oscillator (Eq. 3) is performed to understand how scaling of ground motion intensity and SDOF oscillator yield displacement and yield strength are related. Following Makris and Black (2004), we replace an arbitrary ground motion excitation $\ddot{u}_g$ with a sinusoidal pulse defined by an acceleration intensity $a_p$ and a pulse period $T_p$. Then we restate Eq. 3 by substituting the natural frequency $\omega$ with $\sqrt{S_{a,y}/S_{d,y}}$ as follows:

40 $$\begin{aligned} \ddot{\mu } + 2 \zeta \sqrt{\frac{S_{a,y}}{S_{d,y}}} {\dot{\mu }} + \frac{{\tilde{f}}_s(\mu ) S_{a,y}}{S_{d,y}} = \frac{a_p \sin (2\pi t / T_p)}{S_{d,y}} \end{aligned}$$

In this equation, we identify 7 variables and 2 reference dimensions (length and time). Dimensional analysis using Buckingham’s $\Pi$ theorem gives five dimensionless terms:

41 $$\begin{aligned} \Pi _1 = \mu \;,\; \Pi _2 = \zeta \;,\; \Pi _3 = {\tilde{f}}_s(\mu ) \;,\; \Pi _4 = \frac{S_{a,y}}{a_p} \;,\; \Pi _5 = \frac{S_{d,y}}{a_p T_p^2} \end{aligned}$$

These dimensionless $\Pi$ terms govern the relations between the 7 variables in Eq. 40 and enable the following dimensional analysis.

Scaling the magnitude of the ground motion excitation $$a_p$$ and the yield point of the SDOF oscillator $$(S_{d,y}$$, $$S_{a,y})$$ by the same factor, while keeping the pulse period $$T_p$$, and the behavior parameters of the SDOF oscillator $${\tilde{f}}_s(\mu )$$ and $$\zeta $$ constant, will lead to the same response displacement ductility $$\Pi _1 = \mu $$ since $$\Pi _2$$ to $$\Pi _5$$ are kept constant. However, scaling $$a_p$$ and scaling $$S_{a,y}$$ to keep $$R^*$$ constant, while preserving $$S_{d,y}$$, means that the $$\Pi _5$$ term changes (Eq. 41). Therefore, $$\Pi _1 = \mu $$ changes, leading to a different displacement ductility response of the SDOF oscillator. In order to preserve the same displacement ductility of the SDOF oscillator as the magnitude of the ground motion excitation $$a_p$$ changes, the yield point of the SDOF oscillator $$(S_{d,y}$$, $$S_{a,y})$$ must move along the radial line in the YPS defined by the period of the SDOF oscillator *T*, mimicking the scaling of SDOF oscillator yield point in the CP framework (Fig. 8a). If the SDOF oscillator yield point scales by the same factor as the ground motion excitation magnitude, its CYD strength reduction factor $$R^*$$ and displacement ductility $$\mu $$ will be preserved. The dimensional analysis illustrated in Fig. 8b shows the original and the scaled SDOF oscillator yield points.

The conducted dimensional analysis, and the $\Pi _5$ term in particular, indicate that the yield point of the SDOF oscillator and the intensity of the considered seismic hazard (specified by an elastic design spectrum) must scale consistently in order to preserve dimensional consistency of the yield SDOF oscillator governing Eq. 3, as indicated by Buckingham’s $\Pi$ terms shown in Eq. 41. Next, we show how such consistent scaling can be applied to the ductility-strength $\mu$-$R^*$-$S_{d,y}$ and strength-ductility $R^*$-$\mu$-$S_{d,y}$ relations derived in the CYD framework.

### Scaling of the ductility-strength relations in the CYD framework

**Fig. 9:**
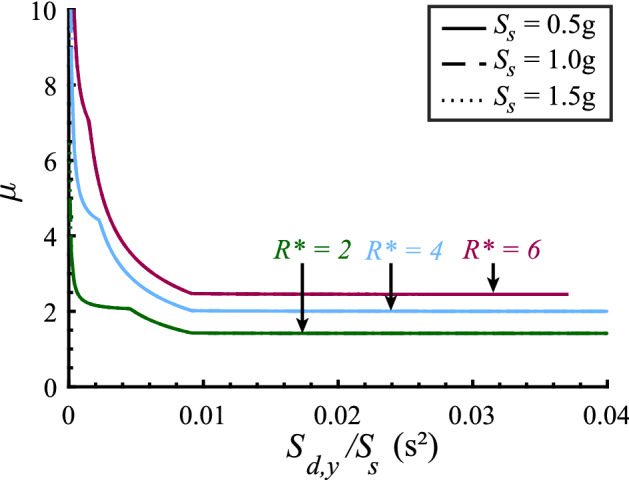
Normalized $\mu$-$R^*$-$S_{d,y}/S_{s}$ relation for different SDOF oscillator displacement ductility values $\mu$ and different seismic hazard intensities $S_s$, given $T_c = 0.6s$

Equations 11, 16 and 23 that define the $\mu$-$R^*$-$S_{d,y}$ ductility-strength relation feature the ratio of the SDOF oscillator yield displacement $S_{d,y}$ to spectral acceleration $S_{s}$ that defines the elastic design spectrum associated with the magnitude of the seismic hazard. We adopt this ratio to normalize the abscissa of the $\mu$-$R^*$-$S_{d,y}$ relation graphs shown in Fig. 6. The graphs shown in Fig. 9 are comparable to the $\mu$-$R^*$-$S_{d,y}$ ductility-strength relation graphs shown in Fig. 6. The normalized $\mu$-$R^*$-$S_{d,y}/S_{s}$ relation graphs overlap completely for different seismic hazard intensities. Therefore, the normalized $\mu$-$R^*$-$S_{d,y}/S_{s}$ relation is independent of seismic hazard intensity. Notably, this relation still depends on the elastic design spectrum corner period $T_c$.

### Scaling of the strength-ductility relations in the CYD framework

**Fig. 10:**
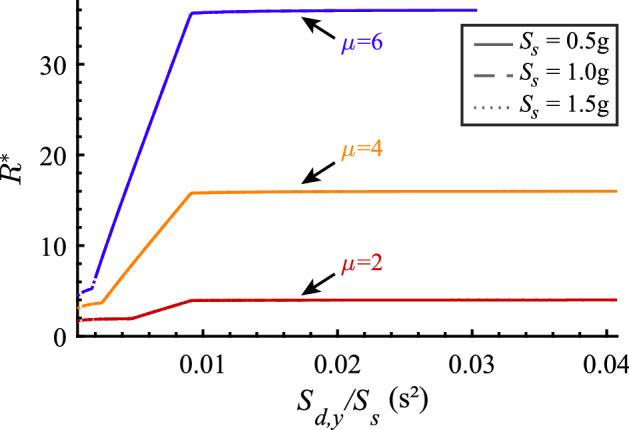
Normalized $R^*$-$\mu$-$S_{d,y}/S_{s}$ relation for different SDOF oscillator displacement ductility values $\mu$ and different seismic hazard intensities $S_s$ and $T_c = 0.6s$

Equations 29, 32 and 37 that define the $R^*$-$\mu$-$S_{d,y}$ strength-ductility relation also feature the ratio $S_{d,y}/S_s$. Thus, we adopt this ratio to normalize the abscissa of the $R^*$-$\mu$-$S_{d,y}$ relations shown in Fig. 7. The outcome is shown in Fig. 10. The normalized $R^*$-$\mu$-$S_{d,y}/S_{s}$ relation graphs overlap completely for different seismic hazard intensities. Therefore, the normalized $R^*$-$\mu$-$S_{d,y}/S_{s}$ relation is independent of hazard intensity. Yet, because of the corner period $T_c$, they are not completely independent of the seismic hazard.

### Length scaling

The $S_{d,y}/S_s$ ratio, derived from the $\Pi _5$ term in Eq. 41, normalizes the CYD framework ductility-strength and strength-ductility relations to make them independent of hazard intensity. This important feature parallels the hazard independence of the strength-ductility *R*-$\mu$-*T* relations in the CP framework. Notably, the variable in *R*-$\mu$-*T* relations is the vibration period of the SDOF oscillator *T*, a time dimension quantity, that can be easily measured for existing or estimated for new structures. However, even if *R*-$\mu$-*T* relations are hazard-independent, they are not dimensionally consistent. For this reason, several authors normalized the SDOF oscillator vibration period *T* by a characteristic period of the ground motion, which is related to the soil type associated with the earthquake ground motion records utilized in their investigations (Ruiz-García and Miranda 2003; Cuesta et al. 2003). Herein, we do not normalize the SDOF oscillator vibration period *T*. Therefore, the dimension of the $S_{d,y}/S_s$ ratio is time squared and the derived ductility-strength and strength-ductility relations are functions of the corner period $T_c$. More important, it is difficult to associate the $S_{d,y}/S_s$ ratio with a measurable characteristic of the surrogate SDOF oscillator or its prototype structure. The $\Pi _5$ term in Eq. 41 is, in fact, a ratio of the characteristic length of the SDOF oscillator (its yield displacement) and the pulse length (Makris and Black 2004). To represent the $\Pi _5$ term, we introduce the characteristic length ratio $\kappa$ (Eq. 8). The characteristic length $\kappa$ features the $S_{d,y}/S_s$ ratio and introduces the corner period (as $T_c^2$) to normalize its time dimension. Thus, $\kappa$ is dimensionless. Notably, $\kappa$ (Eq. 8) can be expressed as a ratio of easily obtainable and physically relevant quantities. The characteristic length of the seismic hazard is the product of the short-period spectral acceleration $S_s$ to the corner period $T_c=S_1/S_s$. The characteristic length of the SDOF oscillator is a product of the yield strain of its yielding material $\varepsilon _y$ and its overturning-moment-equivalent height *H* (Equation 13.2.9 of Chopra, 2016), or reciprocally, the yield displacement normalized by the aspect ratio.

**Fig. 11:**
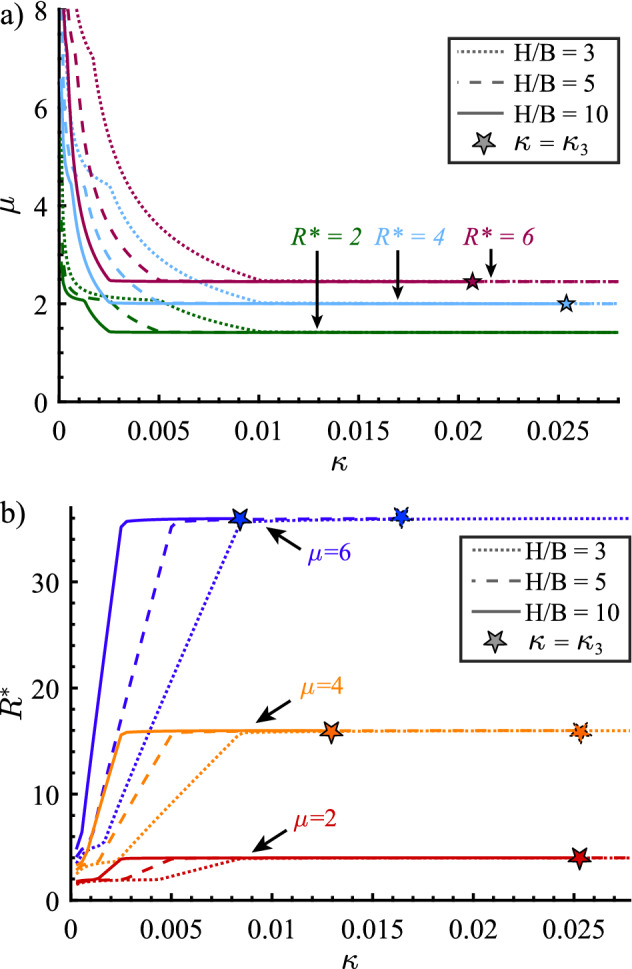
**a** $\mu$-$R^*$-*H*/*B*-$\kappa$ and **b** $R^*$-$\mu$-*H*/*B*-$\kappa$ relations for different $R^*$, $\mu$ and *H*/*B* values, $T_c = 0.6s$ and $T_d = 12s$

The graphs of the $\mu$–$R^*$–$H/B-\kappa$ and $R^*$–$\mu$–$H/B-\kappa$ relations are shown in Fig. 11. These relations are independent of hazard intensity, as the dimensionless characteristic length ratio $\kappa$ preserves the $\Pi _5$ term from Eq. 41. A family of ductility-strength and strength-ductility relations is generated for different values of the SDOF oscillator aspect ratio *H*/*B*, representing the kinematics of the yield mechanism of the prototype structure.

As the characteristic length ratio $\kappa$ increases, $R^*$ tends to $\mu ^2$ from below (or $\mu$ approaches $\sqrt{R^*}$ from above). Thus, the equal displacement rule $C_R \rightarrow 1$ is expressed as $R^*=\mu ^2$ in the CYD framework, analogous to $R=\mu$ in the CP framework.

However, the case limits $\kappa _3$ (Eq. 22), shown in Fig. 11, indicate that Case 3 ceases to apply. Namely, SDOF oscillators with characteristic length ratios $\kappa > \kappa _3$ do not yield or do not not attain the specified, target, ductility. Therefore, strictly, there is no seismic design solution. In case of seismic evaluation, the displacement ductility $\mu$ attained by the SDOF oscillator can be back-calculated using Eq. 27.

Lastly, as the characteristic length ratio $\kappa$ approaches zero, the CYD strength reduction factor $R^*$ values in the $R^*$-$\mu$-*H*/*B*-$\kappa$ relation approach 1, while the displacement ductility values $\mu$ in the $\mu$-$R^*$-*H*/*B*-$\kappa$ relation tend to infinity. Such trends in the CYD framework are consistent with similar trends exhibited by the *R*-$\mu$-*T* relation in the CP framework, as they represent the dynamics of a rigid body that does not deform and does not have a yield displacement.

## Implications of the CYD ductility-strength and strength-ductility relations

The $R^*$-$\mu$-*H*/*B*-$\kappa$ relation shown in Fig. 11, as well as the $R^*$-$\mu$-$S_{d,y}/S_{s}$ (Fig. 10) relation are independent of the seismic hazard intensity. That makes them universal, master relations for seismic design in the CYD framework. Their role is, thus, analogous to that of the *R*-$\mu$-*T* relations in the CP seismic design framework.

There is, however, an important difference between the CP and the CYD frameworks. The CP framework is based on maintaining the vibration period of the SDOF oscillator constant as it transitions from elastic to inelastic seismic response. In that sense, the CP framework is rooted in the dynamics of elastic SDOF oscillators. In contrast, the CYD framework is based on maintaining the yield displacement of the SDOF oscillator, and is thus rooted in the dynamics of inelastic (specifically, elastic-perfectly-plastic) SDOF oscillators.

The advantage of using the CYD framework for seismic design is in an improved interpretation of the inelastic seismic behavior of structures, achieved by referring to their seismic behavior category, size and material yield strain. SDOF oscillators with large $\kappa$ values will remain elastic or develop small displacement ductility, more so if they are slender. Such structures are tall and slender and/or are exposed to low seismic hazard. Thus, their design is not likely to be governed by the seismic design requirements (other than the minimum seismic resistance ones), but by other loads and/or serviceability requirements. Conversely, SDOF oscillators with small $\kappa$ values will undergo significant plastic deformation, necessitating adequately large strength to control the induced displacement ductility.

One disadvantage of the presented CYD framework is its incomplete seismic hazard independence. Namely, the derived ductility-strength and strength-ductility relations depend on the corner periods $T_c$ and $T_d$ used to specify the simplified elastic seismic design spectrum. These corner periods depend on the type of soil present at the site of the prototype structure. Another disadvantage that affects the use of the presented CYD framework in seismic design practice is that the reference, best available, seismic hazard information is provided with respect to an elastic SDOF oscillator and its vibration period. However, while it is possible to characterize the seismic hazard with reference to an inelastic SDOF oscillator (Bozorgnia et al. 2010), to remain within the CYD framework, such characterization should be done with respect to the yield displacement or the characteristic length of the SDOF oscillator, not its vibration period.

The master ductility-strength and strength-ductility relations were developed in the CYD framework assuming a simplified shape of the elastic seismic design spectrum, adequacy of the $C_R$-*R*-*T* relations (Ruiz-García and Miranda 2007), and realism of the Constant Yield Displacement (CYD) assumption. Closed-form CYD framework relations depend on the equations that define the shape of the elastic seismic design spectrum and the form of the $C_R$-*R*-*T* relation: changes of these preliminary elements will change the resulting ductility-strength and strength-ductility relations and may make them more complex. The CYD assumption implies that the SDOF oscillator responds in bending, constraining the range of possible structures to those with a predominantly flexural seismic response. There are many structures that present different response modes, such as shear, sliding and rocking. Moreover, there are brittle structures that do not develop a substantial inelastic response at all. Our ongoing research aims to extend the CYD seismic design framework to address the aforementioned different response modes of structures.

## Conclusions

In this paper, we derive new relations between the displacement ductility and the yield strength of a SDOF oscillator based on the constant yield displacement framework. The key improvement with respect to the conventional relations between the yield strength and displacement ductility in the constant period framework is that new relations directly address the yield displacement of the SDOF oscillator instead of its fundamental vibration period. In a typical seismic design procedure, the structure is expected to behave inelastically under the design hazard, and the design parameter is its base shear at yield. In each design iteration, the yield displacement remains virtually the same because the mechanical characteristics and the geometry of the structure do not change, while its vibration period changes. As a consequence, the new strength-ductility relations enable a comprehensive seismic design process with fewer iterations, and afford a straightforward interpretation of the inelastic behavior of the structure. Furthermore, the new relations streamline the Yield Point Spectrum design procedure, as they lead to the same results without the need to draw a graph, i.e., calculate the seismic response of all possible SDOF oscillators. Moreover, we perform a dimensional analysis to normalize the new strength-ductility and ductility-strength relations such that they become independent of hazard intensity. These normalized relations are the master relations for seismic design within the constant yield displacement framework. The main goal of this study is the demonstration of the use of these relations for the seismic design of structures. An indicative example showing a potential application of the use of these relations for the seismic evaluation of structures is included in the Appendix of this paper. However, the illustration of the detailed process for the use of these relations for the seismic evaluation of structures is beyond the scope of this paper and will be further investigated in the future. Finally, we outline the steps of the constant yield displacement seismic design procedure and illustrate it using two examples, one conventional and one that features multiple performance objectives.

## Supplementary information

Three Microsoft Excel files are available as supplementary material (Silva et al. 2022). The first spreadsheet calculates the yield spectral acceleration* S*_*a,y*_ of the SDOF oscillator given its yield displacement, its target displacement ductility and an elastic design spectrum. This spreadsheet facilitates seismic design of a new structure in the CYD framework. The second spreadsheet calculates the displacement ductility \(\mu\) given the yield point of the SDOF oscillator and the elastic design spectrum, and facilitates seismic evaluation of an existing structure in the CYD framework. The third spreadsheet calculates a displacement ductility fragility curve and the mean annual frequency of exceeding a given displacement ductility threshold, given the yield point of the structure and a seismic hazard curve for the location of the structure. This spreadsheet facilitates evaluation of the seismic risk for an existing structure in the CYD framework.

## Figures and Tables

**Fig. 12:**
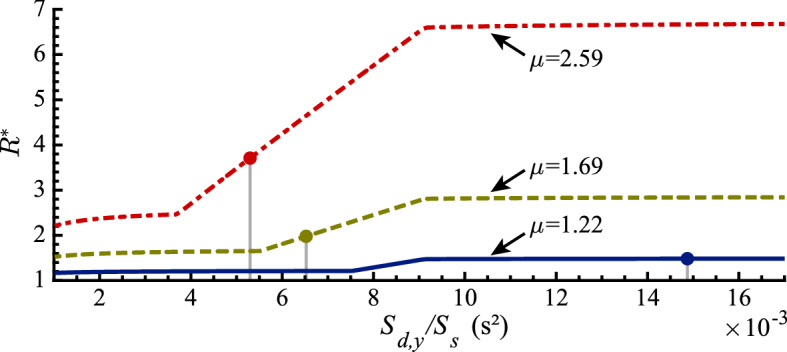
$R^*$-$\mu$-$S_{d,y}/S_{s}$ relation graphs for $T_c = 0.6s$ and $T_d = 12s$ and the three displacement ductility values from Table 1. The circles indicate the design points for each performance objective in Table 2

## Data Availability

No data was used in this paper.

## Appendix A: Seismic evaluation in the CYD framework

The ductility-strength relation is used to evaluate an existing structure with respect to the evaluation-basis seismic hazard as follows:

- Preliminary steps:
  - Specify the seismic hazard values of $S_s$, $S_1$, and $T_d$ to define the evaluation-basis elastic seismic design spectrum. If $T_d$ is not available, select a value several times larger than $T_c = S_1/S_s$.
  - Specify the geometry and mass distribution of the existing structure and the yield strain of the yielding material.
  - Identify the seismic behavior category of the existing structure and estimate its displacement ductility capacity.
- Step 1: Define the SDOF oscillator surrogate of the existing structure by computing yield spectral acceleration $S_{a,y}$ and yield displacement $S_{d,y}$. This is accomplished by performing a pushover analysis and a linearization of the pushover curve to define its yield point (De Luca et al. 2013) or following the linearization procedures described in the building codes (CEN 2005; FEMA 2000).
- Step 2: Check if the SDOF oscillator is in the inelastic range for this hazard by calculating $S_{d,y,max}$ using Eq. 20. If $S_{d,y} > S_{d,y,max}$, the SDOF oscillator is in the elastic range for this hazard and the next steps may be skipped.
- Step 3: Determine the case thresholds $S_{d,y1}$ and $S_{d,y2}$ (Eqs. 10 and 15):
  - If $S_{d,y} < S_{d,y1}$ then it is Case 1: $R^* = S_s / S_{a,y}$. Calculate the evaluation-basis displacement ductility $\mu$ using Eq. 11.
  - If $S_{d,y1}< S_{d,y} < S_{d,y2}$ then it is Case 2 and $R^* = S_s / S_{a,y}$. Calculate the evaluation-basis displacement ductility$\mu$ using Eq. 16.
  - If $S_{d,y} > S_{d,y2}$, then, assume it is Case 3, calculate $R^* = S_s^2 T_c^2 / (4 \pi ^2 S_{a,y} S_{d,y})$ and the evaluation-basis displacement ductility $\mu$ using Eq. 23.
- Step 3: Check for Cases 4, 5 and 6 by calculating $S_{d,y3}$ using Eq. 21.
  - If $S_{d,y} < S_{d,y3}$, it is not case 4, 5 or 6.
  - If $S_{d,y}> S_{d,y3}$, then back-calculate the evaluation-basis displacement ductility $\mu$ using Eq. 26.
- Step 5: Compare the displacement ductility capacity of the SDOF oscillator to the evaluation-basis displacement ductility $\mu$ to conclude if the existing structure is satisfactory or not.

The presented procedure for seismic evaluation of an existing structure in the CYD framework is similar to the one in Tsiavos and Stojadinović (2019). It is also similar to the CP seismic evaluation procedures (e.g., Example 7.2 in Chopra, 2016). The principal difference between the seismic evaluation procedures in the CYD and CP frameworks is the need to determine the yield displacement versus the vibration period of the SDOF oscillator surrogate of the existing structure.

### Seismic evaluation example

The building examined in Hernández-Montes and Aschheim (2019) is used herein. The seismic hazard, as described in the seismic design example in the main body of this paper, is defined by: $S_s = 1.0\hbox {g}$, $T_c = 0.6\hbox {s}$ and $T_d = 12\hbox {s}$. Herein, we perform the evaluation of the initial design of this four-story reinforced concrete moment-resisting frame building from Hernández-Montes and Aschheim (2019), following the steps described above:

- Step 1: The pushover analysis of the building indicates that its yield displacement $u_y = 0.09m$ and its base shear force at yield $V_y = 5377.9 kN$. Thus, the SDOF oscillator yield displacement $S_{d,y} = u_y / \Gamma = 0.09 / 1.36 = 0.068m$ and yield spectral acceleration $S_{a,y} = V_y / (W \cdot \alpha ) = 5377 / (33628 \cdot 0.63) = 0.254 g$.
- Step 2: $S_{d,y,max} = 1.79m$, thus the SDOF oscillator yields.
- Step 3: $S_{d,y1} = 0.02\,m$ and $S_{d,y2} = 0.09\,m$, thus $S_{d,y1}< S_{d,y} < S_{d,y2}$, meaning that the SDOF oscillator is in Case 2. Then $R^* = S_s / S_{a,y}$ = 3.94. Using Eq. 16, the evaluation-basis displacement ductility $\mu = 2.34$ ($\theta _2 = 79.12$).
- Step 4: $S_{d,y3} = 0.76m$. Therefore, the SDOF oscillator is not in Case 4, 5 or 6.
- Step 5: The evaluation-basis displacement ductility $\mu = 2.34$ is lower than the displacement ductility capacity of the building $\mu = 2.4$. Thus, the building fulfills its design objective.

## Appendix B: Graphical performance-based seismic design in the CYD framework

It is straightforward to graphically perform the performance-based seismic design task for the same shear wall building exemplified in the main body of this paper (Table 2). This process involves computing the $S_{d,y}/S_s$ ratio values for the three performance objectives and finding the three design points on $R^*$-$\mu$-$S_{d,y}/S_{s}$ master design graphs Fig. 10) parameterized by the target displacement ductility values for each performance objective, as shown in Fig. 12. The advantage of using these master seismic design graphs is that the design points corresponding to different performance objectives are simple to draw on the same graph. However, the disadvantage is that the governing performance objective is not immediately identifiable. Namely, after identifying the performance-based design points, one has to convert the graphically obtained strength reduction factors $R^*$ into the yield spectral accelerations $S_{a,y}$ for each performance objective using the corresponding elastic design spectra and the appropriate case of the CYD design procedure (Cases 1, 2 or 3). Fortunately, the cases are easily differentiated by the inflection points of the $R^*$-$\mu$-$S_{d,y}/S_{s}$ master relation graphs.

## Appendix C: Seismic risk assessment in the CYD framework

Ruiz-García and Miranda (2007) characterized the empirical cumulative probability distribution of the ratio of the maximum inelastic and elastic SDOF oscillator displacements $C_R$ as lognormal with central tendency ${\tilde{C}}_R$ (Eq. 4) and the dispersion equal to ${\tilde{\sigma }}_{\ln {(C_R)}}$ (Eq. 5). Further, the displacement ductility of the SDOF oscillator $\mu =R \cdot C_R$ (Chopra 2016). This information is used to develop SDOF oscillator displacement ductility fragility curves given its yield point ($S_{d,y}$, $S_{a,y}$). However, the uncertainties associated with the yield point of the SDOF oscillator are not considered herein.

The parameters of the $C_R$ lognormal probability distribution are given in the CP framework, in terms of the CP strength reduction factor *R* and the vibration period of the SDOF oscillator $T = 2 \pi \sqrt{S_{d,y}/S_{a,y}}$. Furthermore, the seismic hazard information for a particular site is commonly provided with reference to $S_a(T)$, the elastic spectral acceleration value for a vibration period *T*. It is, therefore, convenient to translate the specification of the SDOF oscillator from the CYD framework to the CP framework.

Given a SDOF oscillator with a yield point $(S_{d,y}, S_{a,y})$ and an elastic design spectrum associated with a certain seismic hazard intensity (i.e., $R^*$), the expressions for calculating SDOF oscillator *T* and *R* are given in Table 3 for each case defined during the derivation of the $\mu$-$R^*$-$S_{d,y}$ relation.

**Table 3:** Equations for calculating SDOF oscillator period *T* and CP strength reduction factor *R* from its yield point $(S_{d,y}, S_{a,y})$ and CYD strength reduction factor $R^*$

Case	*T*	*R*
1	$2\pi \sqrt{S_{d,y}R^*/S_{s}}$	$R^*$
2	$2\pi \sqrt{S_{d,y}R^*/S_{s}}$	$T_c \sqrt{S_{s} R^*} / ( 2 \pi \sqrt{S_{d,y}})$
3	$4\pi ^2 S_{d,y} \sqrt{R^*} / (S_{s}T_c)$	$\sqrt{R^*}$
4,5,6^a^	$T_d$	$\sqrt{R^*}$ or $\mu$

^a^ Using $$R^* =R^*_{min}$$, thus $$T = T_d$$

Recalling $\mu = R \cdot C_R$ and noting that *T* and *R* are deterministic functions of $S_{d,y}$ and $R^*$ of the SDOF oscillator, the lognormal probability distribution of SDOF oscillator displacement ductility for a given seismic hazard intensity is defined by the mean:

42 $$\begin{aligned} \ln ({\tilde{\mu }}) = \ln (R) + \ln ({\tilde{C}}_R) \end{aligned}$$

and the standard deviation:

43 $$\begin{aligned} \sigma _{\ln ({\tilde{\mu }})} = \sigma _{\ln {({\tilde{C}}_R)}} \end{aligned}$$

Thus, the lognormal probability distribution of SDOF displacement ductility $\mu$ can be computed directly from the lognormal probability distribution of $C_R$ given by Ruiz-García and Miranda (2007). The caveat that the ground motions used by Ruiz-García and Miranda (2007) adequately represent the seismic hazard at the location of the structure remains.

Varying the seismic hazard intensity changes the elastic design spectrum. Given a SDOF oscillator with a yield point $(S_{d,y}, S_{a,y})$ and period $T = 2 \pi \sqrt{S_{d,y}/S_{a,y}}$, the variation of seismic hazard intensity is reduced to the variation of the strength reduction factor $R^*$ and, consequently, variation of *R* per Table 3. Thus, probability distributions of the SDOF oscillator displacement ductility $\mu$ can be calculated for different seismic hazard intensities.

These probability distributions are the basis for assessing the seismic risk of a given SDOF oscillator. Namely, a displacement ductility fragility curve, which states the exceedance probability of a displacement ductility threshold conditioned on the intensity of the seismic hazard (preferably stated in terms of $S_a(T)$), is computed by sampling the displacement ductility probability distributions at different seismic hazard intensities for the given displacement ductility threshold. Further, given a seismic hazard curve (preferably that of the mean annual frequency of $S_a(T)$), the seismic risk is computed by convolving it with the displacement ductility fragility curve (Tsiavos et al. 2021a). Therefore, the mean annual frequencies for the relevant range of displacement ductility thresholds are obtained. Interestingly, the Yield Frequency Spectra (Vamvatsikos and Aschheim 2016) are the mean annual frequencies of different displacement ductility values for SDOF oscillators defined by their yield point $(S_{d,y}, S_{a,y})$.

Calculations of displacement ductility probability distributions, displacement ductility fragility and seismic risk curves require a programmable computer. Thus, they are implemented in a spreadsheet (Silva et al. 2022).

## Code availability

Three Microsoft Excel spreadsheets are available online (Silva et al. 2022) as an electronic supplement to this paper.
